# New approach to the development of tailor-made feed for fish larvae using Zebrafish *Danio rerio* as a model

**DOI:** 10.1371/journal.pone.0326665

**Published:** 2025-06-24

**Authors:** Giovanni S. Molinari, Michal Wojno, Macdonald Wick, Karolina Kwasek

**Affiliations:** 1 Center for Fisheries, Aquaculture, and Aquatic Sciences, Southern Illinois University, Carbondale, Illinois, United States of America; 2 Department of Animal Science, The Ohio State University, Columbus, Ohio, United States of America; 3 Department of Biological Sciences, University of New Hampshire, Durham, New Hampshire, United States of America; Tanta University Faculty of Agriculture, EGYPT

## Abstract

Protein hydrolysates have been used extensively as dietary protein for larval fish. Typically, they are expensive, difficult to produce, and show varying results when utilized for different species. This study proposed a practical hydrolysis method that utilizes endogenous enzymes within the body to “auto-hydrolyze”, or digest its tissue proteins with its own endogenous enzymes, and produce a fishmeal tailored to nutritional requirements and absorptive capacity of larval fish. The objectives for this experiment were to determine the: 1) effect of the proposed hydrolysis method on tissue protein breakdown level; and 2) effect of dietary inclusion of obtained hydrolysates on larval growth performance, using Zebrafish (*Danio rerio*). Whole-body adult Zebrafish were utilized to produce an unhydrolyzed fishmeal, and three fishmeals hydrolyzed for 1, 2, and 3 h, respectively. Three diets were formulated, defined by their dietary protein supply. The Unhydro diet contained unhydrolyzed Zebrafish meal. The 50% Hydro diet contained 50% Zebrafish meal hydrolysates and 50% unhydrolyzed Zebrafish meal. The 100% Hydro diet contained 100% Zebrafish meal hydrolysates. Five groups were utilized in this study, with three groups receiving one of the produced Zebrafish meal-based diets. Larvae fed a commercial starter diet and *Artemia*, respectively, were included as reference groups. Larval fish were randomly stocked into tanks (100 fish per tank) at 3 days-post-hatch (dph), and the trial was carried out until 18 dph. Electrophoretic analysis showed that the proposed hydrolysis method was able to efficiently hydrolyze the protein within Zebrafish body. The feeding trial results found no significant differences in weight, total length, or survival between the Unhydro, 50% Hydro, and 100% Hydro groups. The proposed hydrolysis method provides a practical and potentially cost-effective approach to producing species-specific fishmeal hydrolysates. Further research is necessary to determine whether the produced hydrolysates can improve the growth of larval fish in other species.

## Introduction

To achieve more control over the nutritional input during the larval stage, and provide more optimal dietary protein, extensive research has centered on producing formulated diets to reduce the use of live feeds [1–7]. Generally, intact marine protein sources have been used in these formulated larval diets [1,8–10], with fishmeal (FM) being the most widely used [11,12]. Commercial FM has been traditionally produced from whole fish, typically small, oily species, or wastes originating from fish processing. Fishmeal is attractive as a protein source because of its high protein content, high digestibility, complete amino acid profile and high palatability [13–15]. Fishmeal also serves as a partial source of vitamins and minerals, and although the majority of lipids are extracted, the remaining lipids (5–10%) contain highly unsaturated essential fatty acids [11,16]. Positive results on the early introduction of FM-based diets have been observed in larval rearing, with most successes occurring in co-feeding protocols, where dry diets are fed along with live feed [17–19].

Still, there are limitations to the ability of FM-based diets to fully replace live feeds at first feeding [20–22]. For example, the complex proteins that are present in FM are not efficiently digested by larval fish, especially during the first feeding stage [20]. Many fish species raised in aquaculture are altricial, not possessing fully developed digestive tracts at the start of exogenous feeding [21]. The underdeveloped digestive tracts lead to reduced digestive capacity of altricial larval fish [21,22], and as a result, the utilization of protein hydrolysates has been extensively studied [7,21,23–25]. Providing hydrolyzed protein in a diet for larval fish helps account for the reduced activity of proteolytic enzymes in the underdeveloped digestive tract, by breaking down the protein to smaller peptides and free amino acids (FAA) prior to ingestion [26]. Altricial larvae have been found to have an increased assimilation efficiency of these peptides and FAA compared to intact protein [22,27]. While the inclusion of protein hydrolysates has led to improved utilization of formulated diets in some species [7,22,28–30], the full replacement of live feeds has remained elusive in the rearing of many commercial species.

In larval fish, amino acids are required at high levels for tissue synthesis and serve as the major source of energy [31–33]. The requirement levels for amino acids, the indispensable amino acids (IDAA) in particular, vary among species, and ages of fish within the same species [34]. While specific IDAA requirements are unknown for larval fish [35], the amino acid profile of same-species muscle has been proposed to represent the amino acid requirement for larvae [36–38]. Given this premise, and the known variations in amino acid profiles of FM produced from different species [16], perhaps the species-specific FM obtained from adult fish would provide the right amino acid profile for fish larvae. This would be beneficial for the advancement of formulated dry diets in larval rearing, with the species-specific FM potentially serving as a dietary protein source better suited to replace live feed, compared to current commercial starter diets. Additional species-specificity can be added to incorporate the previously mentioned benefits of hydrolyzed FM, using endogenous enzymes to mimic *in vivo* digestion [39,40].

An *in vitro* hydrolysis method utilizing same-species endogenous enzymes was developed in Kwasek et al. [41], and later tested in tandem with same-species muscle in Molinari et al. [42]. Specifically, the endogenous enzymes and muscle of adult Largemouth Bass (*Micropterus salmoides*) were used to produce a protein hydrolysate that served as a species-specific dietary protein source for first-feeding Largemouth Bass larvae [42]. Additionally, the use of species-specific enzymes and muscle was shown to increase growth in larval Walleye (*Sander vitreus*), compared to protein produced from non-species-specific sources [43].The endogenous enzymes utilized in those studies were extracted from the harvested digestive tracts through homogenization and centrifugation, then mixed with isolated muscle. In this study, we proposed a modified and simplified method of protein hydrolysis that removes the individual extractions of muscle and digestive tracts and produces a species-specific FM from whole fish. This method has not previously been tested in any other studies as a means of producing dietary protein for larval fish.

The aim of this study was to develop a more optimal protein source for larval fish and to advance the production of formulated diets to replace live feeds. The objectives were to; 1) determine the effect of the proposed simplified *in vitro* hydrolysis method on tissue protein breakdown level and the production of a species-specific hydrolyzed FM; and 2) evaluate the effect of dietary inclusion of the obtained FM hydrolysate on larval fish. The performance of the hydrolysates was assessed based on growth and survival, the gene expression of the intestinal peptide transporter PepT1, and the postprandial muscle FAA pool, used as an indicator of dietary amino acid availability. This study utilized Zebrafish (*Danio rerio*), as they have been found to be an ideal model species for nutritional research [44].

## Materials and methods

### Experimental conditions

The feeding trial was conducted at the Center for Fisheries, Aquaculture, and Aquatic Sciences at Southern Illinois University-Carbondale (SIUC), IL. The experiment was carried out in strict accordance with the recommendations in the Guide for the Care and Use of Laboratory Animals of SIUC. The SIUC Institutional Animal Care and Use approved all of the protocols performed (protocol #21−006). The experiment was carried out using a semi-recirculated aquaculture system (Iwaki Aquatic, Holliston, MA, USA). The system was equipped with two mechanical filters, a carbon filter, a UV light, and a biofilter. The photoperiod was maintained with overhead lights and the lights were on from 08:00–18:00 for 10 h of light and 14 h of darkness. The illumination provided was at 245 lux and the distance between the surface of the water and the light source was 10 cm. The inflow was set at 100 mL/min for each tank. During the study, the temperature was 27.37°C (± 0.62) and the pH was 7.25 (± 0.23). The salinity of the system was kept at 1–3 ppt to prolong the viability of the live feed [45].

### Zebrafish hydrolysis

The hydrolysis method for this study was based on the *in vitro* method described in Kwasek et al. [41], with modification. Briefly, adult Zebrafish were kept at 27°C and fed to satiation one hour before harvesting. The fish were euthanized with an excess of tricaine methanesulfonate (MS-222) (Sigma-Aldrich, St. Louis, MO, USA) with a dose of 0.4 mg/mL. The whole bodies were ground three times with a meat grinder (General Food Service, Weston, FL, USA) and were stored on ice before and after each run through the grinder. After dilution with deionized water (1:2), the fish mince was homogenized with a PowerGen 1000 (Fisher Scientific, Waltham, MA, USA) tissue homogenizer on high speed for 10 min, at room temperature. The tissue to digestive tract ratio of the harvested Zebrafish was roughly 6:1. The homogenates were moved to containers (12 L), placed in a water bath at 27°C and stirred using an overhead stirrer (VWR VOS 16, VWR, Radnor, PA, USA) for the duration of the hydrolysis. The pH was adjusted to 7–9 using sodium bicarbonate for the entire hydrolysis to mimic the intestinal digestion of Zebrafish. Four Zebrafish meals were produced, three hydrolysates that were hydrolyzed for 1, 2, and 3 h, respectively, and an unhydrolyzed Zebrafish meal. After the hydrolysis, the solution was brought to 90°C for 15 min to stop any enzymatic activity. The unhydrolyzed Zebrafish meal was immediately brought to 90°C after homogenization to prevent any hydrolysis. While this meal is not a completely intact product, it is referred to as “unhydrolyzed” to clearly differentiate it from the products that underwent the controlled hydrolysis process. All products were frozen at −20°C and subsequently freeze-dried to remove moisture. After freeze-drying, lipids were extracted from each product using the Soxhlet method, with diethyl ether as the solvent [46]. Electrophoretic analysis of both unhydrolyzed and hydrolyzed products was conducted at The Ohio State University (Columbus, OH).

### Diets

Three diets were formulated to be isonitrogenous and isolipidic and meet the essential nutrient requirements of larval fish [9]. They were formulated to contain 51% crude protein, and 14% crude lipids (Table 1), and analyzed compositions were obtained after production (Table 2). The Unhydro diet did not contain any of the hydrolyzed Zebrafish meal and was solely based on the unhydrolyzed Zebrafish meal. The 50% Hydro diet was based on 50% Zebrafish hydrolysate mix and 50% unhydrolyzed Zebrafish meal. The 100% Hydro diet was 100% based on the Zebrafish meal hydrolysates for dietary protein. The hydrolysate mix contained equal parts of the 1, 2, and 3 h hydrolysates. This mix was used to provide a wide range of protein fragments in the diet.

**Table 1:** Diet formulations for Zebrafish larvae feeding experiment.

Ingredients (%)	Unhydro	50% Hydro	100% Hydro
Unhydrolyzed Zebrafish meal	74.00	37.00	–
Hydrolysate Mix^a^	–	38.75	78.00
Starch	4.85	3.10	0.85
Krill Meal^b^	5.00	5.00	5.00
Fish oil^c^	4.00	4.00	4.00
Lecithin^d^	4.00	4.00	4.00
Mineral mix^e^	3.00	3.00	3.00
Vitamin mix^f^	3.00	3.00	3.00
CaHPO_**4**_	1.00	1.00	1.00
Taurine	1.00	1.00	1.00
Choline chloride	0.10	0.10	0.10
Vitamin C^g^	0.05	0.05	0.05
Sum	100	100	100

^a^Equal mixture of 1, 2, and 3 h hydrolyzed Zebrafish meals

^b^Processed *Euphausia superba* (Florida Aqua Farms, Dade City, FL, USA).

^c^Cod liver oil (MP Biomedicals, Solon, OH, USA).

^d^Refined soy lecithin (MP Biomedicals, Solon, OH, USA).

^e^Bernhart-Tomarelli mineral mix with 5 ppm selenium in a form of sodium selenite (Dyets, Bethlehem, PA, USA).

^f^Custom Vitamin Mixture (mg/kg diet) Thiamin HCl, 6.84; Riboflavin, 7.2; Pyridoxine HCl, 10.29; Niacin, 16.35; D-Calcium Pantothenate, 75.84; Folic Acid, 1.89; D-Biotin, 0.24; Vitamin B12 (0.1%), 30; Vitamin A Palmitate (500,000 IU/g), 14.49; Vitamin D3 (400,000 IU/g), 12.39; Vitamin E Acetate (500 IU/g), 198; Menadione Sodium Bisulfite, 3.54; Inositol, 750 (Dyets, Bethlehem, PA, USA).

^g^L-Ascorbyl-2-Polyphosphate (Argent Aquaculture, Redmond, WA, USA).

**Table 2:** Analyzed composition of formulated diets.

Analyzed Composition (g/100g) Dry Matter	Unhydro	50% Hydro	100% Hydro
Crude Protein (N x 6.25)	48.14 (± 0.18)	48.19 (± 0.09)	48.26 (± 0.17)
Crude Lipids	10.04 (± 0.08)	10.07 (± 0.14)	10.06 (± 0.12)
Ash	17.92 (± 0.04)	17.69 (± 0.05)	17.49 (± 0.03)
Alanine	2.94 (± 0.03)	2.88 (± 0.02)	2.90 (± 0.02)
Arginine	2.81 (± 0.01)	2.67 (± 0.04)	2.64 (± 0.02)
Aspartic Acid	4.32 (± 0.03)	4.26 (± 0.03)	4.24 (± 0.01)
Cysteine	0.46 (± 0.00)	0.46 (± 0.00)	0.48 (± 0.00)
Glutamic Acid	6.32 (± 0.15)	6.16 (± 0.06)	6.20 (± 0.08)
Glycine	2.92 (± 0.03)	2.80 (± 0.03)	2.93 (± 0.03)
Histidine	1.20 (± 0.00)	1.17 (± 0.01)	1.11 (± 0.02)
Hydroxyproline	0.58 (± 0.01)	0.55 (± 0.02)	0.61 (± 0.00)
Isoleucine	2.24 (± 0.01)	2.23 (± 0.02)	2.17 (± 0.02)
Leucine	3.56 (± 0.02)	3.52 (± 0.03)	3.45 (± 0.02)
Lysine	3.77 (± 0.03)	3.72 (± 0.03)	3.72 (± 0.01)
Methionine	1.20 (± 0.01)	1.17 (± 0.01)	1.19 (± 0.00)
Phenylalanine	2.01 (± 0.02)	1.99 (± 0.02)	1.94 (± 0.04)
Proline	2.17 (± 0.02)	2.12 (± 0.02)	2.18 (± 0.03)
Serine	1.86 (± 0.07)	1.79 (± 0.04)	1.83 (± 0.05)
Taurine	1.64 (± 0.25)	1.58 (± 0.03)	1.42 (± 0.05)
Threonine	1.97 (± 0.01)	1.94 (± 0.02)	1.93 (± 0.01)
Tryptophan	0.45 (± 0.01)	0.42 (± 0.01)	0.42 (± 0.00)
Tyrosine	1.52 (± 0.00)	1.44 (± 0.10)	1.50 (± 0.01)
Valine	2.42 (± 0.01)	2.40 (± 0.03)	2.35 (± 0.02)
Sum	46.37 (± 0.37)	45.27 (± 0.40)	45.21 (± 0.21)

Diets were analyzed in triplicates and the values are presented as mean (± S.D).

### Experimental design

Larvae were produced from broodstock obtained from a local pet store (Petco, Carbondale, IL). The fish were separated by sex for two weeks prior to breeding. After this period, the broodstock were combined in a 2:1 ratio of females to males and a wire mesh (1.5 mm) was added to the breeding tank with an artificial plant to induce spawning. The fish were allowed to breed for 24 h and then removed. The eggs hatched after approximately 48 h.

At 3 days-post-hatch (dph), larval Zebrafish were randomly distributed into 15 (3 L) tanks, with 100 fish per tank. There were five treatment groups in this study, with three replicate tanks each. The first group, **LF**, was a live feed reference group, which received only live feed for the duration of the study. The LF group was fed rotifers (3–7 dph) and then switched to *Artemia* nauplii (7–18 dph), with a transition period from 7 to 11 dph where both types of live feed were provided. The next group, **Com**, was a dry feed reference group, which received a commercial starter diet (Otohime, Marubeni Nisshin Feed Co., Tokyo, Japan). The last three groups corresponded to each of the three diets produced utilizing the Zebrafish meal. These were the **Unhydro**, **50% Hydro**, and **100% Hydro** groups. The fish were fed in excess and, to ensure constant feed availability, fish received the feed every hour from 08:00–18:00. The size of the feeds added to the tanks were < 150 μm. The tanks were siphoned twice daily to prevent the degradation of water quality due to the build-up of waste and uneaten feed. The experiment was carried out until 18 dph.

### Sampling and measuring

At the conclusion of the study (18 dph), all fish from each tank were individually weighed and the total length measured. Samples for additional analysis were also taken at the conclusion of the study. Three fish from each tank were euthanized with an overdose of MS-222 and stored in RNAlater (Invitrogen by Fisher Scientific, Waltham, WA, USA) for the analysis of PepT1 gene transcription in the gut. Samples for PepT1 expression were taken 2 and 24 h after feeding to analyze postprandial and fasting levels, respectively. Additionally, two sets of whole-body samples were taken from each tank, with five fish per sample. The sampled fish were euthanized in liquid nitrogen and stored at −80°C for further FAA analysis. These sets of samples were also taken 2 and 24 h after feeding and represented postprandial and basal FAA levels, respectively.

### PepT1 analysis

PepT1 analysis was conducted using the same method described in Molinari et al. [47]. The digestive tracts were removed from the sampled Zebrafish. The samples were processed using TRIzol Reagent (Ambion, Foster City, CA, USA) and RNA was extracted and purified using the On-Column PureLink DNase Treatment (PureLink^™^ RNA Mini Kit and PureLink DNase, Invitrogen, Carlsbad, CA, USA) following the manufacturer’s instructions. Once purified, the ng/μL of each RNA sample was obtained using a spectrophotometer (Nanodrop OneC, Thermo Fisher Scientific, Waltham, MA, USA). From this point, 2 μg of RNA from each sample was reverse transcribed using the High-Capacity cDNA Reverse Transcription Kit (Applied Biosystems, Foster City, CA, USA) to obtain a 20 μL cDNA solution. The cDNA solutions were then added to a tube with 380 μL of water, to produce the cDNA sample for each tank. Gene expression of each cDNA sample was measured using a Bio-Rad (Hercules, CA, USA) CFX Opus 96 Real-Time PCR System. Each qPCR reaction mixture (20 μL) contained 9 μL of cDNA sample, 10 μL of PowerUp SYBR Green Master Mix (Thermo Fisher Scientific, Waltham, MA, USA), and 0.5 μL of 800 nmol each of forward and reverse primers. Primers were synthesized by Integrated DNA Technologies (Coralville, IA, USA). The primers are listed in Table 3. Each qPCR reaction was run in technical duplicates. The qPCR cycle consisted of 95°C for 10 min, followed by 40 cycles of 95°C for 20 seconds and 60°C for 35 seconds, followed by a melting curve to ensure the amplification of only a single product in each well. Relative gene expression was calculated using the 2ΔΔCt method, normalizing the target gene expression to the expression of *ef1a* (reference gene).

**Table 3:** Primers used for PepT1 analysis.

Gene	GenBank Accension #	Primers	Function	Reference
PepT1/*slc15a1*	AY300011	F: ATCATTGTGCTCATCGTGGC	Di- and tripeptide absorption	[48]
		R: GGCGAAGATGATGCTCACAG		
*ef1a*	NM_131263	F: TTGAGAAGAAAATCGGTGGTGCTG	Reference gene	[49]
		R: GAACGGTGTGATTGAGGGAAATTC		

### Free amino acid analysis

Free amino acid analysis of fish muscle was performed according to the method described in Kwasek et al. [41]. The muscle from the whole-body fish samples were obtained by removing the head, caudal fin, and viscera. Muscle samples of three fish from each tank were combined and homogenized together with 0.1 mol/L HCl in 1:9 (w/v) and spun at 12,000 × g (4°C, 15 min). Supernatants were collected, filtered (Milipore, 10 kDa cutoff at 15,000 × g, 4°C, 30 min), and later diluted with 0.1 mol/L HCl (1:19 v/v) containing norvaline and sarcosine (40 μmol/L) as internal standards. Blanks (0.1 mol/L HCl + 40 μmol/L norvaline and sarcosine) and external standards (Sigma acid/neutral and basic amino acids) were prepared along with the sample preparation. The same concentration of glutamine in 0.1 mol/L HCl as an external standard was prepared and added to the basic amino acid standard. Free amino acids were quantified using Shimadzu Prominence Nexera—i LC-2040C Plus (Shimadzu, Japan) according to the Shimadzu protocol No. L529 with modifications. Free amino acid concentrations (expressed as μmol/g wet body weight) were calculated in LabSolutions software version 5.92 (Shimadzu, Japan) using internal and external standards.

### SDS-PAGE

Sodium dodecyl sulfate-polyacrylamide gel electrophoresis (SDS-PAGE) was performed to visualize the products of tissue hydrolysis. Electrophoretic analysis of the samples was conducted on a discontinuous, reducing 12.5% T, SDS polyacrylamide gel with modifications of the method described by Updike et al. [50]. Briefly, samples were homogenized in dissociation buffer (8 mol urea, 2 mol thiourea, 60 mmol Tris buffer, pH 6.8, containing 3% SDS, 350 mmol DTT, and 0.002% bromophenol blue) overnight at 25°C with agitation and diluted with dissociation buffer as needed. Approximately 10 μL of sample (10 mg/mL) dilutions were loaded onto each lane with a 3% stacking gel containing 1% SDS. The proteins were resolved at 150 V cm^-1^ until the dye front reached the bottom of the gel. The gel was stained with Coomassie Brilliant Blue dye (40% methanol, 5% acetic acid, 0.04% Coomassie Brilliant Blue G-250) overnight and destained with 10% acetic acid. The destained gel was scanned on an AZURE c600 scanner (Azure Biosystems, Dublin, CA, USA).

### Statistical analysis

Statistical analysis was run using R version 3.5.2 (Boston, MA, USA) [51]. The weight and total length measurements, along with survival data, were analyzed using one-way ANOVA and a Tukey test to detect significant differences between groups. The same statistical analysis was applied to the FAA data collected, with a one-way ANOVA and a Tukey test being used to detect significant differences in FAA levels between groups within each set of samples (2 and 24 h). Lastly, the data analyzed for PepT1 expression are presented as average fold change in relation to the LF reference group, however the one-way ANOVA and Tukey test were conducted on the dCt values within each set of samples (2 and 24 h). In the case of statistical analysis conducted on all data collected, differences between groups were considered significant at p < 0.05.

## Results

### Hydrolysis of zebrafish tissue

**Fig 1:**
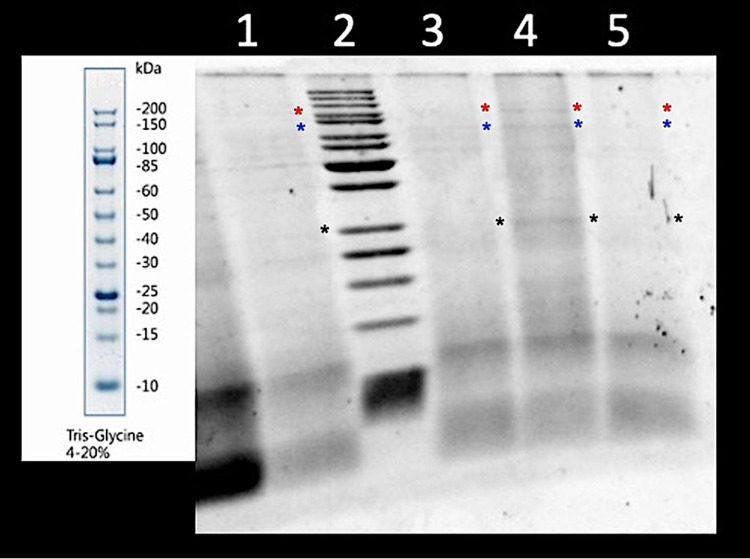
Representative reducing 12.5% T SDS-PAGE of Zebrafish protein products. Lanes (1) 2 h hydrolysate; (2) broad range molecular weight standard (200–10 kDa); (3) 1 h hydrolysate; (4) unhydrolyzed; (5) 3 h hydrolysate. Colored asterisks marked protein bands in the unhydrolyzed sample that are observed to disappear in the hydrolysate samples.

The results of the Zebrafish meal hydrolysis were obtained utilizing 12.5% T SDS-PAGE (Fig 1). The products added into each lane were: Lane (1) 2 h hydrolysate; (2) broad range molecular weight standard (200–10 kDa); (3) 1 h hydrolysate; (4) unhydrolyzed; (5) 3 h hydrolysate. The gel shows that the proposed method was able to efficiently hydrolyze the protein within the Zebrafish body and produce a hydrolyzed FM. The results show that as the hydrolysis progressed, from unhydrolyzed to the 1, 2, and 3 h hydrolysates, there appear to be decreasing levels of larger protein fragments (> 50 kDa) and increasing levels of smaller proteins (< 25 kDa), and small peptides lower than 10 kDa. Evidence of hydrolysis can be observed by the disappearance of protein bands at higher molecular weights in the hydrolysate samples, that are seen in the unhydrolyzed meal. The bands are seen at ~150 kDa (red asterisk), ~ 100 kDa (blue asterisk), and ~50 kDa (black asterisk) in Lane 4, containing the unhydrolyzed Zebrafish meal. However, the bands only appear very faintly or not at all in lanes 1, 3, and 5, which contain the varying degrees of hydrolyzed Zebrafish meal.

### Growth and survival

The LF group showed significantly higher average weight (p < 0.001) and total length (p < 0.001) than the other groups (Table 4). Additionally, the Com group had a significantly higher average total length than the Unhydro, 50% Hydro, and 100% Hydro groups (p < 0.001), and a significantly higher average weight than both the 50% and 100% Hydro groups (p < 0.001). The 100% Hydro group had a significantly lower survival, compared to the LF group (p = 0.045). There were no significant differences in growth or survival among the Unhydro, 50% Hydro, and 100% Hydro groups.

**Table 4:** Diet effect on growth and survival.

Group	Avg. Weight (mg)	Avg. Total Length (mm)	Survival (%)
**LF**	8.53^c^ (± 0.57)	9.77^c^ (± 0.32)	90.67^b^ (± 5.03)
**Com**	1.98^b^ (± 0.21)	4.99^b^ (± 0.21)	80.67^ab^ (± 3.06)
**Unhydro**	1.18^ab^ (± 0.10)	4.16^a^ (± 0.18)	76.33^ab^ (± 1.16)
**50% Hydro**	0.98^a^ (± 0.02)	4.16^a^ (± 0.32)	76.67^ab^ (± 4.16)
**100% Hydro**	0.96^a^ (± 0.20)	3.84^a^ (± 0.41)	65.00^a^ (± 19.70)

Values are presented as means (± S.D.). Superscript letters indicate statistically significant differences between groups. The significance was determined using a One-Way ANOVA and a Tukey test with a p value < 0.05.

### PepT1 expression

**Fig 2:**
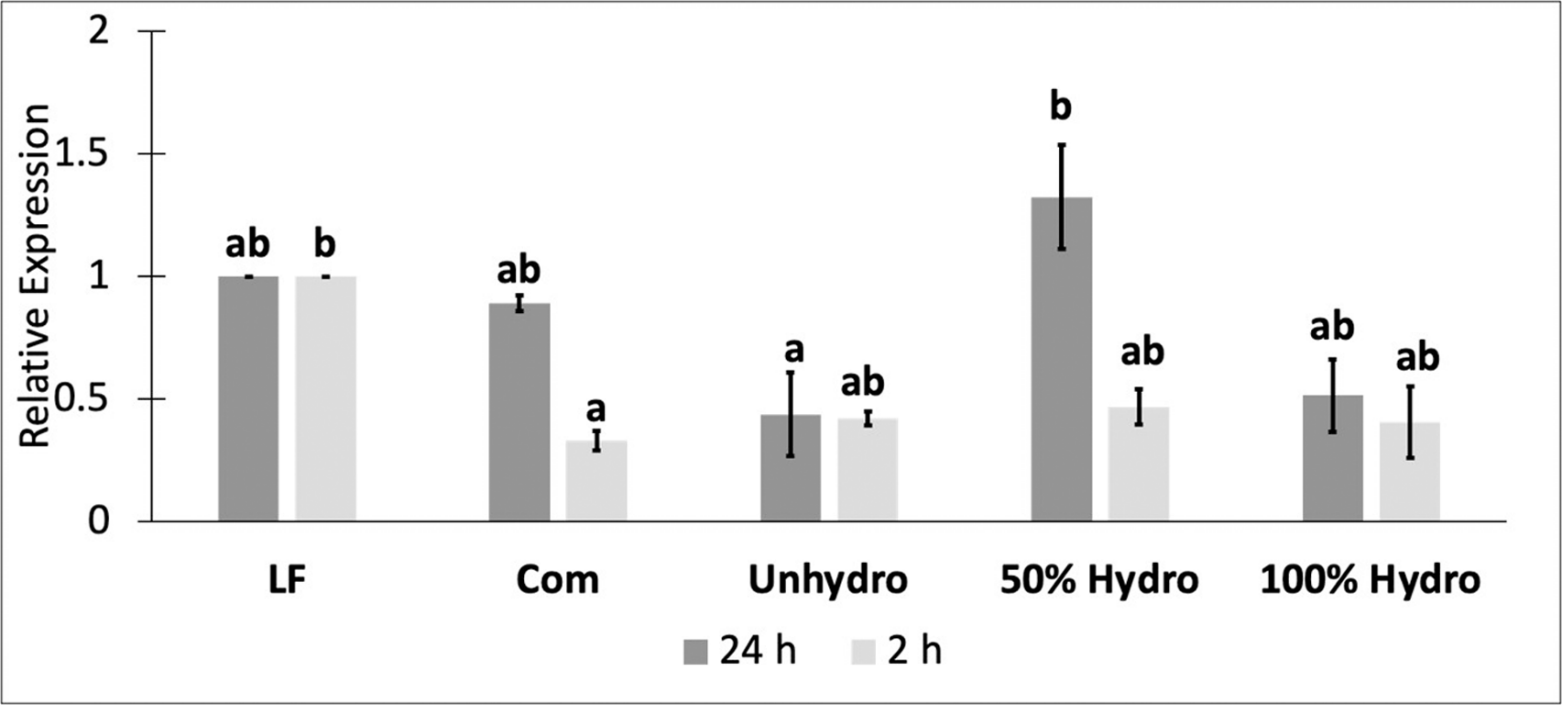
PepT1 expression. Values are presented as mean fold change in relation to LF group, and error bars represent standard error. The letters indicate significant statistical differences (p < 0.05) among 24 h and 2 h expression. The significance was determined using a One-Way ANOVA and a Tukey test.

At 24 h after feeding, the 50% Hydro group had a significantly higher expression of PepT1, compared to the Unhydro group (p = 0.038) (Fig 2). No other significant differences were observed between the 24 h expressions of PepT1 of the remaining groups. At 2 h after feeding, the Com group expressed significantly lower levels of PepT1 than the LF group (p = 0.049). No other significant differences were observed between the 2 h expressions of PepT1 of the remaining groups.

### Muscle free amino acid composition

The Com group had significantly higher postprandial levels of total FAA and free IDAA in the muscle than all other groups (p < 0.001) (Table 5). The LF group had significantly higher postprandial levels of total FAA and free IDAA than the three groups that received the Zebrafish meal (p < 0.001), and the 100% Hydro had significantly lower levels than all of the groups. Both the LF and Com groups had significantly higher postprandial levels of free dispensable amino acids (DAA) in the muscle than the other groups (p < 0.001), and the 100% Hydro group had significantly lower levels of DAA in the muscle, compared to the other four groups.

**Table 5:** Postprandial FAA compositon in muscle.

Amino Acid (μmol/g)	LF	Com	Unhydro	50% Hydro	100% Hydro
**Aspartic Acid**	1.58^a^ (± 0.06)	2.21^ab^ (± 0.70)	1.97^ab^ (± 0.04)	2.91^b^ (± 0.38)	2.17^ab^ (± 0.26)
**Glutamic Acid**	3.54^b^ (± 0.20)	4.11^c^ (± 0.04)	2.17^a^ (± 0.26)	1.86^a^ (± 0.15)	1.74^a^ (± 0.16)
**Asparagine**	0.87^c^ (± 0.13)	0.89^c^ (± 0.04)	0.42^b^ (± 0.09)	0.21^a^ (± 0.01)	0.27^ab^ (± 0.01)
**Serine**	1.67^b^ (± 0.17)	1.85^b^ (± 0.01)	1.00^a^ (± 0.13)	0.89^a^ (± 0.02)	0.78^a^ (± 0.03)
**Glutamine**	3.56^c^ (± 0.13)	3.21^c^ (± 0.05)	1.73^b^ (± 0.20)	1.37^ab^ (± 0.15)	1.25^a^ (± 0.09)
**Histidine**	3.22^d^ (± 0.18)	4.72^e^ (± 0.10)	2.22^c^ (± 0.26)	1.56^b^ (± 0.08)	1.00^a^ (± 0.06)
**Glycine**	3.10^d^ (± 0.15)	3.75^e^ (± 0.12)	2.29^c^ (± 0.22)	1.62^b^ (± 0.05)	0.96^a^ (± 0.09)
**Threonine**	1.48^ab^ (± 0.11)	4.11^d^ (± 0.11)	1.91^c^ (± 0.20)	1.82^bc^ (± 0.16)	1.13^a^ (± 0.09)
**Arginine**	0.82^b^ (± 0.14)	1.05^c^ (± 0.04)	0.51^a^ (± 0.04)	0.59^a^ (± 0.07)	0.46^a^ (± 0.03)
**Alanine**	4.94^b^ (± 0.43)	4.72^b^ (± 0.02)	1.98^a^ (± 0.19)	1.74^a^ (± 0.05)	1.44^a^ (± 0.08)
**Tyrosine**	6.45^d^ (± 0.15)	4.96^c^ (± 0.34)	2.75^b^ (± 0.32)	1.42^a^ (± 0.53)	0.71^a^ (± 0.04)
**Lysine**	1.95^c^ (± 0.26)	2.91^d^ (± 0.07)	1.13^b^ (± 0.10)	1.06^ab^ (± 0.08)	0.74^a^ (± 0.04)
**Methionine**	3.01^c^ (± 0.06)	3.61^d^ (± 0.07)	1.13^b^ (± 0.12)	1.10^b^ (± 0.14)	0.79^a^ (± 0.05)
**Valine**	1.15^c^ (± 0.12)	1.37^d^ (± 0.03)	0.61^ab^ (± 0.04)	0.67^b^ (± 0.08)	0.46^a^ (± 0.03)
Cysteine *Not detected	–	–	–	–	–
**Tryptophan**	0.19^a^ (± 0.08)	0.34^c^ (± 0.01)	0.21^ab^ (± 0.01)	0.32^bc^ (± 0.04)	0.31^bc^ (± 0.02)
**Phenylalanine**	0.42^a^ (± 0.06)	0.67^b^ (± 0.02)	0.38^a^ (± 0.05)	0.41^a^ (± 0.03)	0.32^a^ (± 0.02)
**Isoleucine**	0.73^b^ (± 0.13)	1.33^c^ (± 0.03)	0.66^ab^ (± 0.04)	0.73^b^ (± 0.08)	0.52^a^ (± 0.04)
**Leucine**	0.89^ab^ (± 0.13)	2.18^c^ (± 0.04)	1.06^b^ (± 0.05)	1.13^b^ (± 0.12)	0.77^a^ (± 0.05)
**Proline**	2.51^d^ (± 0.33)	1.64^c^ (± 0.07)	0.94^b^ (± 0.12)	0.72^b^ (± 0.08)	0.20^a^ (± 0.01)
**IDAA**	13.84^c^ (± 1.03)	22.28^d^ (± 0.38)	9.84^b^ (± 0.90)	9.40^b^ (± 0.77)	6.51^a^ (± 0.36)
**DAA**	28.22^c^ (± 1.63)	27.35^c^ (± 1.10)	15.26^b^ (± 1.52)	12.75^b^ (± 0.19)	9.51^a^ (± 0.75)
**TFAA**	42.06^c^ (± 2.66)	49.63^d^ (± 1.47)	25.10^b^ (± 2.42)	22.15^b^ (± 0.89)	16.02^a^ (± 1.11)

The values presented are postprandial levels, 2 h after feeding. Values are presented as means (± S.D.). Superscript letters indicate statistically significanct differences between groups. The significance was determined using a One-way ANOVA and a Tukey test with a p value < 0.05. Indispensable amino acids (IDAA) = Ile, Leu, Lys, Met, Phe, Thr, Trp, Val, Arg, and His. Dispensable amino acids (DAA) = Ala, Asp, Asn, Glu, Gln, Gly, Pro, Ser, and Tyr. TFAA = total free amino acids.

Significant differences were observed among the postprandial levels of individual FAA as well. The 50% Hydro group had a significantly higher level of free aspartic acid in the muscle, 2 h after feeding compared to the LF group (p = 0.017). The postprandial levels of free glutamic acid and free arginine were significantly higher in the Com group compared to all other groups (p < 0.001; p < 0.001), and the LF group had significantly higher levels of both amino acids than the 3 groups that received the Zebrafish meal. The 50% Hydro group presented a significantly lower level of free asparagine than all other groups (p < 0.001), and the Unhydro group had a level significantly lower than both the LF and Com groups. The postprandial levels of free serine and free alanine in the muscle were significantly lower in the three groups that received the Zebrafish meal (p < 0.001; p < 0.001), compared to the LF and Com groups. The measured levels of free glutamine were significantly higher in the LF and Com groups, compared to all other groups (p < 0.001), and the Unhydro group had a significantly higher level than the 100% Hydro group. Significant differences between each group were observed for both free histidine and free glycine levels in the muscle (p < 0.001; p < 0.001). The highest levels were observed in the Com group, followed by the LF, Unhydro, 50% Hydro, and 100% groups, respectively. The 100% Hydro group had significantly lower levels of free threonine than all other groups (p < 0.001), and the Com group had significantly higher levels than all other groups. The LF group had a significantly higher level of free tyrosine in the muscle 2 h after feeding than all other groups (p < 0.001), and the Com group also had a significantly higher level than the three Zebrafish meal groups. The Unhydro group had a significantly higher level of free tyrosine than the 50% and 100% Hydro groups (p < 0.001). The postprandial levels of free lysine, free methionine, and free valine were significantly higher in the Com group compared to all other groups (p < 0.001; p < 0.001; p < 0.001), and the levels of all three amino acids were significantly higher in the LF group compared to the Unhydro, 50% Hydro, and 100% Hydro groups. For free lysine, the level was significantly lower in the 100% Hydro group compared to the Unhydro group (p < 0.001). The level of free methionine was significantly higher in the Unhydro and 50% Hydro groups than the 100% Hydro group (p < 0.001). The 50% Hydro group had a significantly higher level of free valine, compared to the 100% Hydro group (p < 0.001). Both the LF and Unhydro groups had significantly lower levels of free tryptophan in the muscle, compared to the Com group (p = 0.003). The level of free phenylalanine in the muscle 2 h after feeding was significantly higher in the Com group than all other groups (p < 0.001). The Com group also had significantly higher levels of free isoleucine and free leucine, compared to all other groups (p < 0.001; p < 0.001). Both the LF and 50% Hydro groups had significantly higher levels of free isoleucine than the 100% Hydro group (p < 0.001). The 100% Hydro group showed a significantly lower level of free leucine, compared to the Unhydro and 50% Hydro group (p < 0.001). Lastly, the LF group had a significantly higher level of free proline in the muscle than all other groups (p < 0.001), and the Com group had a significantly higher level than the three groups that received the Zebrafish meals. The 100% Hydro group had a significantly lower level of free proline than all other groups (p < 0.001). The results for all basal levels of FAA analyzed are presented in Table 6.

**Table 6:** Basal FAA compositon in muscle.

Amino Acid (μmol/g)	LF	Com	Unhydro	50% Hydro	100% Hydro
**Aspartic Acid**	1.08 (± 0.22)	1.64 (± 0.07)	1.94 (± 0.03)	2.37 (± 0.03)	2.44 (± 0.21)
**Glutamic Acid**	2.28 (± 0.60)	2.11 (± 0.13)	1.29 (± 0.14)	1.82 (± 0.04)	2.07 (± 0.07)
**Asparagine**	0.52 (± 0.05)	0.40 (± 0.04)	0.15 (± 0.02)	0.11 (± 0.01)	0.29 (± 0.00)
**Serine**	0.43 (± 0.09)	0.59 (± 0.01)	0.35 (± 0.04)	0.67 (± 0.03)	0.64 (± 0.04)
**Glutamine**	2. 00 (± 0.57)	1.30 (± 0.08)	0.81 (± 0.09)	1.02 (± 0.01)	1.18 (± 0.05)
**Histidine**	2.46 (± 0.29)	3.17 (± 0.15)	1.10 (± 0.13)	1.49 (± 0.02)	1.48 (± 0.07)
**Glycine**	3.11 (± 0.21)	2.61 (± 0.14)	1.27 (± 0.17)	1.37 (± 0.02)	1.52 (± 0.07)
**Threonine**	0.26 (± 0.09)	2.06 (± 0.08)	0.88 (± 0.07)	1.15 (± 0.04)	0.93 (± 0.07)
**Arginine**	0.12 (± 0.05)	0.27 (± 0.01)	0.22 (± 0.02)	0.52 (± 0.03)	0.40 (± 0.02)
**Alanine**	2.51 (± 0.38)	1.93 (± 0.09)	1.07 (± 0.11)	2.03 (± 0.05)	1.75 (± 0.10)
**Tyrosine**	5.17 (± 1.27)	4.10 (± 0.37)	0.67 (± 0.08)	1.77 (± 0.45)	2.50 (± 0.14)
**Lysine**	0.35 (± 0.12)	0.74 (± 0.03)	0.39 (± 0.04)	0.82 (± 0.05)	0.63 (± 0.04)
**Methionine**	2.25 (± 0.53)	2.61 (± 0.08)	1.03 (± 0.11)	0.92 (± 0.02)	0.92 (± 0.01)
**Valine**	0.46 (± 0.08)	0.59 (± 0.04)	0.33 (± 0.03)	0.39 (± 0.01)	0.40 (± 0.02)
Cysteine *Not detected	–	–	–	–	–
**Tryptophan**	0.07 (± 0.04)	0.28 (± 0.01)	0.24 (± 0.03)	0.14 (± 0.00)	0.33 (± 0.01)
**Phenylalanine**	0.09 (± 0.02)	0.25 (± 0.01)	0.18 (± 0.03)	0.27 (± 0.02)	0.26 (± 0.02)
**Isoleucine**	0.11 (± 0.02)	0.23 (± 0.01)	0.19 (± 0.02)	0.33 (± 0.02)	0.30 (± 0.02)
**Leucine**	0.20 (± 0.05)	0.40 (± 0.02)	0.34 (± 0.03)	0.63 (± 0.04)	0.48 (± 0.03)
**Proline**	0.16 (± 0.21)	0.17 (± 0.02)	0.01 (± 0.00)	0.16 (± 0.00)	0.06 (± 0.01)
**IDAA**	6.39 (± 1.25)	10.60 (± 0.41)	4.90 (± 0.49)	6.66 (± 0.10)	6.14 (± 0.29)
**DAA**	17.25 (± 3.41)	14.84 (± 0.74)	7.56 (± 0.66)	11.32 (± 0.53)	12.45 (± 0.65)
**TFAA**	23.63 (± 4.63)	25.44 (± 1.15)	12.46 (± 1.15)	17.98 (± 0.63)	18.59 (± 0.93)

The values presented are basal levels, 24 h after feeding. Values are presented as means (± S.D.). Indispensable amino acids (IDAA) = Ile, Leu, Lys, Met, Phe, Thr, Trp, Val, Arg, and His. Dispensable amino acids (DAA) = Ala, Asp, Asn, Glu, Gln, Gly, Pro, Ser, and Tyr. TFAA = total free amino acids.

## Discussion

Given the difficulties in quantifying amino acid requirements for larval fish [34,35], the approach utilized in this study served as an attempt at a practical proof of concept for the long-held notion that a species’ amino acid requirements match the amino acid profile of the same-species tissue [36–38,52]. Both ethical and biosecurity considerations were taken into account when applying this approach, which involved feeding species-specific protein to larvae. Natural cannibalistic behavior observed in both farmed and wild populations of many fish species [53,54] provides support that research utilizing this method falls within ethical boundaries. Additionally, the heat treatment applied for both the unhydrolyzed and hydrolyzed Zebrafish meal produced not only deactivated the enzymatic activity, but served to eradicate any potential infectious agents present in the adult Zebrafish tissue. This method adhered to guidelines for production of protein products from farmed fish [55,56] and prevented disease transfer from the adult Zebrafish to the larvae. Research based on this concept is an important step towards understanding larval fish nutritional requirements, particularly as it relates to their amino acid requirements that can ultimately lead to development of optimal species-specific diets.

The first main objective of this study was to assess the efficacy of the proposed hydrolysis method on tissue protein breakdown level and the production of a species-specific hydrolyzed FM. The efficacy of the hydrolysis process was visualized using electrophoretic analysis (SDS-PAGE), showing the disappearance of larger protein fragments in hydrolyzed meals. The electrophoretic results suggest the proposed method was able to hydrolyze the tissue proteins in the Zebrafish body and produce protein/peptide products with a higher proportion of low molecular weight proteins than the unhydrolyzed product. The increased proportion of these low molecular weight proteins/peptides was intended to provide dietary protein that would be more efficiently absorbed by the larval fish. Studies suggest that small peptides (5–75 kDa) are more efficiently absorbed by larval fish than larger, intact proteins [6,22]. Based on electrophoretic analysis, there appears to be more of these smaller peptides in the hydrolyzed Zebrafish meals, compared to the unhydrolyzed meal, with increasing amounts of peptides < 10 kDa appearing as the time of hydrolysis progressed.

Given the previous success observed with the use of species-specific hydrolysates as a protein source for larval fish [41–43], the hydrolysis method in this study was proposed to create a more practical and applicable approach for the production of these hydrolysates. The major benefits of the proposed hydrolysis method are rooted in its simplicity, reproducibility, and species-specificity. The autolytic basis of this hydrolysis method is similar to that utilized in the production of fish silage, where fish by-products or whole fish are minced, and the tissue is allowed to be autolyzed by proteolytic enzymes contained in both the guts and muscle [57–60]. During the silage process, the endogenous enzymes break down the tissue proteins to produce a liquid with high levels of water-soluble peptides and FAA [61]. Silage production represents a cost-effective and simple way to produce hydrolyzed fish protein. However, this process can be time consuming and presents significant variations in protein recovery and degree of hydrolysis [57,62]. As a result, recent research has focused on enzymatic hydrolysis as a method of producing fish protein hydrolysates. Enzymatic hydrolysis involves the addition of separately obtained proteolytic enzymes to hydrolyze the protein in an incubated system [61,63]. This method provides more control over the peptide profile of the hydrolysate, with the selection of enzymes being based on their cleavage-specificity [64]. There is also greater control over the degree of hydrolysis as the pH and temperature of the method can be adjusted throughout the process to maintain an optimal environment for the activity of the selected enzymes [63]. This ultimately results in a higher degree of hydrolysis in a shorter amount of time, and a more consistent and reproduceable protein profile than silage production [63,64]. A major pitfall to the use of enzymatic hydrolysis is the difficulty in selecting the most ideal enzyme(s) and their high cost [60,65].

The hydrolysis method in this study provides a middle ground between these two production methods, with added benefits of species-specific protein and species-specific enzymes. In our method, whole fish were minced, and the proteins were autolyzed using only the endogenous enzymes. While the digestive enzymes play the most significant role in the autolysis process in fish, other enzymes contained within the body have proteolytic capabilities and contribute to the breakdown of body proteins [61,66].The autolysis was continuously monitored and controlled to maintain a constant temperature and pH, mimicking the *in vivo* digestion of the Zebrafish digestive tract. The *in vitro* hydrolysis method of mimicking *in vivo* digestion with endogenous enzymes has been proven to be effective in this and other studies [41,67,68]. This mimicked *in vivo* digestion produces a dietary protein that is hydrolyzed with the species-specific composition of proteolytic enzymes, representing a molecular weight profile that likely matches that of protein digested within the fish body. It also produces a FM that provides the optimal amino acid profile for the larvae of the same species [52,69]. The continuous control over pH and temperature of this method allows it to be applicable to other species as a means of producing species-specific FM. Although in the present study the pH was maintained to be constantly alkaline to mimic the intestinal digestion of Zebrafish, an agastric species, the same general incubation method was utilized in a gastric species [41]. That study switched the pH of the hydrolysis solution from acidic to alkaline to mimic the movement from stomach (acidic digestion) to intestine (alkaline digestion), and found that the activity of the endogenous enzymes, and thus hydrolysis of the protein, continued after the pH switch [41]. The proposed method provides a practical way to produce hydrolyzed, species-specific FM, where the cost-effectiveness, simplicity, and autolysis aspects of silage production are maintained, and the efficiency and control are improved through monitored incubation.

Another benefit of the presented hydrolysis method is that it produces a hydrolyzed FM out of whole-body fish, instead of just isolated muscle. Not only does this contribute to the simplicity of the method by removing the dissection and isolation of muscle and digestive tracts, it also provides a more sustainable and complete dietary nutrient source. The harvesting of muscle from fish, or filleting fish, leaves behind 50–70% as waste [63,70]. The fillet from fish contains only 15–25% of the total protein in the body [64,71] and leaves behind a fish frame that contains the head, tail, scales, bones, and viscera [72]. These often-discarded parts of the fish contain ~58% protein [72] and can provide additional nutrients including minerals, vitamins, and essential fatty acids [73–75]. Although the levels and effects of these components were not directly assessed in this study, further investigation and modifications to this method could present the potential of the hydrolyzed FM to be a species-specific source of these nutritional components as well. In an industry where future growth hinges on increased sustainability, a novel method that discards over 50% of its materials as waste would be short-lived. Thus, the use of whole fish as a source of highly digestible protein/peptides in FM as described in this study presents a more viable and environmentally conscious approach to produce a species-specific diet for larval fish that has shown positive effects in previous studies [41–43].

The second main objective of this study was to assess the effect of dietary inclusion of the obtained species-specific FM hydrolysate on larval Zebrafish. The LF and Com groups served as benchmarks for growth and physiological responses of Zebrafish under typical laboratory rearing methods [76]. Only the 100% Hydro group exhibited a significant difference in survival, significantly lower than the LF group. The significant reductions in all growth parameters in the dry feed groups relative to the LF group highlight the need for the development of suitable dry diets for larval rearing. Among the dry feed groups, all three of the Zebrafish meal groups showed a significantly lower total length compared to the Com group, and the 50% Hydro and 100% Hydro groups exhibited significant reductions in average weight. While the Com group provides a reference for larval rearing on available commercial starter diets, important variables like dietary composition, ingredients, and physical structure are not controlled for, which limits the ability to make strong conclusions on the dietary effects behind the differences observed. Thus, the most informative comparisons in this study come from the three groups fed with the formulated Zebrafish meal-based diets.

The growth and survival results from this study showed no significant differences in weight, total length, or survival between the Unhydro, 50% Hydro, and 100% Hydro groups, therefore the inclusion of the hydrolyzed FM failed to improve the growth of the larvae. These results are opposite of those seen previously in studies that tested the use of species-specific protein for larval diets and observed a significant increase in growth [41,43]. Additionally, the lack of improvement through the inclusion of hydrolyzed protein in general contradicts results observed in many other larval studies [7,24,28,42]. As mentioned previously, the inclusion of the hydrolyzed Zebrafish meal in the diet was intended to promote increased growth by providing protein in the form of small peptides and FAA. The dietary provision of these small peptides and FAA has been found to increase absorption of dietary protein by larval Walleye [27], and Striped Bass (*Morone saxatilis*) [27], as well as larval Atlantic Halibut (*Hippoglossus hippoglossus*) [77], and Senegalese Sole (*Solea senegalensis*) [6]. The absorption of the di-/tripeptides from the diets was assessed based on the expression of PepT1, an intestinal peptide transporter [78,79]. PepT1 expression has been found to be responsive to different molecular weight profiles of dietary protein [80–82].

At the 2 h sampling time, there was a numerical trend of lower PepT1 expression in the Unhydro, 50% Hydro, and 100% Hydro groups, and a significant downregulation in the Com group compared to the LF group. This highlights a major benefit of live feeds. Live feeds contain high levels of water-soluble protein in low molecular weight form, which makes the dietary protein more bioavailable than that in formulated dry diets [34,83]. This may have resulted in a faster absorption of di-/tripeptides in the LF group and a delayed expression of PepT1 in the dry feed groups. This delay in PepT1 expression has been observed in Rainbow Trout (*Oncorhynchus mykiss*), where the expression of PepT1 was significantly higher 12 h postprandially, compared to 2 h in response to dry diets [84]. Consequently, the 2 h postprandial results showed no significant differences in PepT1 expression between the Unhydro, 50% Hydro, and 100% Hydro groups. However, 24 h after feeding, there was a significant upregulation in PepT1 expression in the 50% Hydro group over the Unhydro group. This might suggest that the 50% inclusion of the hydrolyzed Zebrafish meal significantly increased the absorption of dietary protein in di-/tripeptide form. Previous studies that have shown increases in PepT1 expression in response to dietary peptides also observed these differences at 24 h post-feeding [82,85,86]. In larval Snakehead (*Channa argus*), the 50% inclusion of a protein hydrolysate led to a significant increase in the 24 h PepT1 expression over a diet containing no protein hydrolysates and a diet containing 100% of its dietary protein in hydrolyzed form [82]. These results are similar to the 24 h PepT1 results in the present study, although there was only a numerical decrease in PepT1 expression in our 100% Hydro group compared to the 50% Hydro group. In the larval Snakehead, the significant increase in PepT1 correlated with a significantly higher growth due to the increased absorption of di-/tripeptides [82], however in the present study, there were no significant differences in growth between the Unhydro, 50% Hydro, and 100% Hydro groups. The lack of significant differences in growth between these three groups, and the higher growth on an intact protein-based commercial diet, could suggest that Zebrafish are adapted to dry feeds at first feeding and are able to utilize dietary protein in different molecular forms efficiently for growth. The adaptation to dry feeds may stem from a high level of domestication in Zebrafish.

Domestication in fish has been found to influence the digestive abilities of larvae [87–89]. Research has found that upon introduction of compound diets, domesticated larvae started showing significantly higher growth rates compared to the wild-type larvae [88]. This increased growth rate corresponded with an overall decrease in the activity of most digestive enzymes, but an increase in proteolytic enzymes in the domesticated larvae [88]. This difference in digestive enzyme activity was suggested to be a result of “nutritional monotony”, where domesticated fish had been fed high-protein diets for generations, leading to a potential shift towards higher proteolytic activity within the gut [88]. This phenomenon has also been observed in Gilthead Seabream [89]. These studies provide evidence that domestication in fish species can affect the abilities of larvae to digest and utilize dietary components from formulated feeds over generations.

Additional evidence of the effect of possible Zebrafish domestication on the results in this study and other larval studies may be found in the high survival rates achieved with full live feed replacement. The Unhydro and 50% Hydro diets were able to fully replace live feed in this study without significant reductions in survival, supporting survival rates ~76%. This full replacement of live feed has previously been achieved in the rearing of larval Zebrafish with similar survival levels [23,90–92]. Compared to the low survival levels of complete live feed replacement in commercial species like Largemouth Bass [42], Barramundi (*Lates calcarifer*) [93], Sea Bass (*Dicentrarchus labrax*) [1], and Senegalese Sole [94], the observed success of dry feed-only rearing for Zebrafish presents a stark contrast. The significant difference in survival between Zebrafish and those other commercial species could be due to the varying levels of domestication. Domesticated fish have exhibited higher feed intake and reduced stress in intensive settings, contributing to improved survival over their wild-type counterparts during intensive rearing [87,95–97]. Zebrafish are a popular ornamental and lab-raised model species and are classified at the highest level of domestication [98,99]. Common Carp (*Cyprinus Carpio*) is another cyprinid species within this same level of domestication that has shown high survival of larvae reared on dry diets only [100]. Meanwhile, most commercial species in aquaculture exist at lower levels of domestication [101]. For Carp, this increased domestication is a result of a longer history of culture compared to other commercial species. In contrast, Zebrafish have been commercially cultured for much less time, but still reached the highest domestication level. This is a result of very short generation times in Zebrafish (~2–4 months) [102], which has allowed for genetic changes related to domestication to occur much more rapidly compared to other commercialized species [103]. Thus, the varying degrees of domestication among species may be a factor behind the differences in survival between the live feed replacement studies.

Another plausible reason behind the lack of differences in growth across the Unhydro, 50% Hydro, and 100% Hydro groups, is that the dietary protein profiles of each diet were more similar than they were formulated to be. The Unhydro diet was formulated to provide a dietary protein profile with larger, intact fragments, while the 100% Hydro diet was formulated to provide dietary protein in the form of low molecular weight peptides and FAA from the hydrolyzed Zebrafish meal. The 50% Hydro diet was formulated to be a middle ground between those diets, with half of its dietary protein supplied from the unhydrolyzed Zebrafish meal, and the other half from the mix of hydrolyzed Zebrafish meal. Looking at the results from the SDS-PAGE, it is feasible that our “unhydrolyzed” Zebrafish meal may have undergone unintended and unincubated partial hydrolysis. While the unhydrolyzed Zebrafish meal does contain a higher level of large protein fragments > 25 kDa compared to the hydrolyzed products, there seems to be a fair amount of smaller proteins < 25 kDa present. These small fragments may be a result of unintended autolysis occurring during storage prior to and during the homogenization of whole fish. While the fish were stored on ice during harvest, reducing the post-harvest hydrolysis rate [104], the water used during homogenization was room-temperature. This could have allowed for the enzymes contained within the body to partially hydrolyze the body protein during the 10 min homogenization at room temperature. A partial hydrolysis of our ‘unhydrolyzed’ Zebrafish meal could have reduced the variations in dietary molecular weight profiles among our formulated experimental diets. This likely contributed to reduced differences in growth from occurring among the Unhydro, 50% Hydro, and 100% Hydro diets. This poses a limitation to this method, and further research to prevent unintended hydrolysis from occurring prior to incubation would improve control over the final degree of hydrolysis.

The postprandial levels of FAA in the muscle were significantly lower in the 100% Hydro group, compared to the other groups. While this reduction in postprandial FAA has typically been attributed to excess leaching from hydrolysate-based diets [8,77,105], it is unlikely that this led to the reduced FAA levels in the 100% Hydro group. Precautions like smaller meals in shorter time intervals and 2x daily cleanings were taken to ensure pellets consumed by the larvae had not experienced prolonged exposure in the water column. Additionally, no significant differences in growth or survival were seen among the Zebrafish-meal fed groups, suggesting any potential leaching differences among diets did not significantly affect larval performance. However, the 100% Hydro group was the only group to show a significant reduction in survival relative to the LF group. Previous studies have found that high inclusion levels of hydrolysates have hindered the utilization of dietary protein in larval fish, leading to a rapid absorption and excretion of amino acids, or an oversaturation of FAA transporters that limited absorption [30,106,107]. The supply of amino acids from the diet is critical to support the high levels of tissue synthesis required during larval metamorphosis [34]. Given the significantly reduced survival and postprandial FAA levels, it is likely that the full supply of dietary protein in hydrolyzed form reduced dietary amino acid absorption and utilization to a point that hindered the proper development of the larval Zebrafish and led to a significant decrease in survival of the 100% Hydro group.

## Conclusion

Overall, the results from this study showed that the proposed hydrolysis method was able to efficiently hydrolyze the tissue protein within Zebrafish body. This provides a practical, cost-effective, and sustainable method for producing hydrolyzed species-specific FM, that is applicable to a wide range of species. Due to observed partial hydrolysis of the protein that did not undergo the incubated hydrolysis procedure, further research is recommended to prevent this and provide more control over the hydrolysis level of protein produced with this method. Results from the feeding trial show that the inclusion of the species-specific hydrolysates did not improve the growth of the larvae compared to the unhydrolyzed Zebrafish meal. The growth results paired with Pept1 gene expression potentially indicate Zebrafish larvae as being highly adapted to dry feeds at first feeding and able to utilize dietary protein in different molecular forms efficiently for growth. Future research should explore this novel concept on other, less domesticated, fish species.

## Supporting information

S1 File Analyzed parameters. This file contains the raw data for growth, PepT1 expression, and FAA analysis.
(XLSX)

S2 File Raw SDS-PAGE gel. (TIF)

## References

[1] Cahu C, Infante JZ, Escaffre AM, Bergot P, Kaushik S. Preliminary results on sea bass (Dicentrarchus labrax) larvae rearing with compound diet from first feeding. Comparison with carp (Cyprinus carpio) larvae. Aquaculture. 1998;169(1–2):1–7.

[2] Cahu C, Infante JZ. Substitution of live food by formulated diets in marine fish larvae. Aquaculture. 2001;200(1–2):161–80.

[3] Koven W, Kolkovski S, Hadas E, Gamsiz K, Tandler A. Advances in the development of microdiets for gilthead seabream, Sparus aurata: a review. Aquaculture. 2001;194(1–2):107–21.

[4] Yúfera M, Fernández-Díaz C, Pascual E. Food microparticles for larval fish prepared by internal gelation. Aquaculture. 2005;248(1–4):253–62.

[5] Seiliez I, Bruant JS, Zambonino Infante JL, Kaushik S, Bergot P. Effect of dietary phospholipid level on the development of gilthead sea bream (Sparus aurata) larvae fed a compound diet. Aquac Nutr. 2006;12(5):372–8.

[6] Canada P, Conceicao LE, Mira S, Teodosio R, Fernandes JM, Barrios C. Dietary protein complexity modulates growth, protein utilisation and the expression of protein digestion-related genes in Senegalese sole larvae. Aquaculture. 2017;479:273–84.

[7] Sheng Z, Turchini GM, Xu J, Fang Z, Chen N, Xie R, et al. Functional Properties of Protein Hydrolysates on Growth, Digestive Enzyme Activities, Protein Metabolism, and Intestinal Health of Larval Largemouth Bass (Micropterus salmoides). Front Immunol. 2022;13:913024. doi: 10.3389/fimmu.2022.913024 35928824 PMC9343713

[8] Langdon C. Microparticle types for delivering nutrients to marine fish larvae. Aquaculture. 2003;227(1–4):259–75. doi: 10.1016/s0044-8486(03)00508-8

[9] National Research Council, Division on Earth, Life Studies, Committee on the Nutrient Requirements of Fish, Shrimp. Nutrient requirements of fish and shrimp. Washington, DC: National Academies Press. 2011.

[10] Rahmdel KJ, Falahatkar B. Adaptation of pikeperch (Sander lucioperca) to formulated diets: A review. Fish Aquat Life. 2021;29(1).

[11] Li MH, Robinson EH, Hardy RW. Protein sources for feed. Hoboken, NJ: John Wiley and Sons Inc. 2000.

[12] Nankervis L, Southgate PC. An integrated assessment of gross marine protein sources used in formulated microbound diets for barramundi (Lates calcarifer) larvae. Aquaculture. 2006;257(1–4):453–64.

[13] Miles RD, Chapman FA. The benefits of fish meal in aquaculture diets. EDIS. 2006;2006(12).

[14] Tacon AG, Metian M. Global overview on the use of fish meal and fish oil in industrially compounded aquafeeds: trends and future prospects. Aquaculture. 2008;285(1–4):146–58.

[15] Salin KR, Arun VV, Mohanakumaran Nair C, Tidwell JH. Sustainable aquafeed. Sustainable Aquaculture. 2018. p. 123–51.

[16] Hertrampf JW, Piedad-Pascual F. Handbook on ingredients for aquaculture feeds. New York, NY: Springer Science & Business Media. 2012.

[17] Kolkovski S, Koven W, Tandler A. The mode of action of Artemia in enhancing utilization of microdiet by gilthead seabream Sparus aurata larvae. Aquaculture. 1997;155(1–4):193–205.

[18] Rosenlund G, Stoss J, Talbot C. Co-feeding marine fish larvae with inert and live diets. Aquaculture. 1997;155(1–4):183–91.

[19] Engrola S, Figueira L, Conceição LE, Gavaia PJ, Ribeiro L, Dinis MT. Co-feeding in Senegalese sole larvae with inert diet from mouth opening promotes growth at weaning. Aquaculture. 2009;288(3–4):264–72.

[20] Conceição LEC, Yúfera M, Makridis P, Morais S, Dinis MT. Live feeds for early stages of fish rearing. Aquaculture Res. 2010;41(5):613–40. doi: 10.1111/j.1365-2109.2009.02242.x

[21] Kolkovski S. Digestive enzymes in fish larvae and juveniles—implications and applications to formulated diets. Aquaculture. 2001;200(1–2):181–201.

[22] Tonheim SK, Espe M, Hamre K, Rønnestad I. Pre-hydrolysis improves utilisation of dietary protein in the larval teleost Atlantic halibut (Hippoglossus hippoglossus L.). J Exp Mar Biol Ecol. 2005;321(1):19–34.

[23] Carvalho AP, Araújo L, Santos MM. Rearing zebrafish (Danio rerio) larvae without live food: evaluation of a commercial, a practical and a purified starter diet on larval performance. Aquac Res. 2006;37(11):1107–11.

[24] Srichanun M, Tantikitti C, Kortner TM, Krogdahl Å, Chotikachinda R. Effects of different protein hydrolysate products and levels on growth, survival rate and digestive capacity in Asian seabass (Lates calcarifer Bloch) larvae. Aquaculture. 2014;428:195–202.

[25] Delcroix J, Gatesoupe FJ, Desbruyères E, Huelvan C, Le Delliou H, Le Gall MM. The effects of dietary marine protein hydrolysates on the development of sea bass larvae, Dicentrarchus labrax, and associated microbiota. Aquac Nutr. 2015;21(1):98–104.

[26] Chalamaiah M, Dinesh Kumar B, Hemalatha R, Jyothirmayi T. Fish protein hydrolysates: proximate composition, amino acid composition, antioxidant activities and applications: a review. Food Chem. 2012;135(4):3020–38. doi: 10.1016/j.foodchem.2012.06.100 22980905

[27] Rust MB. Quantitative aspects of nutrient assimilation in six species of fish larvae. University of Washington. 1995.

[28] Kotzamanis YP, Gisbert E, Gatesoupe FJ, Zambonino Infante J, Cahu C. Effects of different dietary levels of fish protein hydrolysates on growth, digestive enzymes, gut microbiota, and resistance to Vibrio anguillarum in European sea bass (Dicentrarchus labrax) larvae. Comp Biochem Physiol A Mol Integr Physiol. 2007;147(1):205–14. doi: 10.1016/j.cbpa.2006.12.037 17306580

[29] Xu H, Mu Y, Zhang Y, Li J, Liang M, Zheng K. Graded levels of fish protein hydrolysate in high plant diets for turbot (Scophthalmus maximus): effects on growth performance and lipid accumulation. Aquaculture. 2016;454:140–7.

[30] Canada P, Engrola S, Conceicao LE, Valente LM. Improving growth potential in Senegalese sole (Solea senegalensis) through dietary protein. Aquaculture. 2019;498:90–9.

[31] Fyhn HJ. First feeding of marine fish larvae: are free amino acids the source of energy?. Aquaculture. 1989;80(1–2):111–20.

[32] Parra G, Rønnestad I, Yúfera M. Energy metabolism in eggs and larvae of the Senegal sole. J Fish Biol. 1999;55:205–14.

[33] Rønnestad I, Tonheim SK, Fyhn HJ, Rojas-García CR, Kamisaka Y, Koven W. The supply of amino acids during early feeding stages of marine fish larvae: a review of recent findings. Aquaculture. 2003;227(1–4):147–64.

[34] Hamre K, Yufera M, Rønnestad I, Boglione C, Conceição LE, Izquierdo M. Fish larval nutrition and feed formulation: knowledge gaps and bottlenecks for advances in larval rearing. Rev Aquac. 2013;5:S26-58.

[35] Jobling M. Fish nutrition research: past, present and future. Aquac Int. 2016;24(3):767–86.

[36] Cole DJA. The amino acid requirements of pigs-the concept of an ideal protein. Pig News Info. 1980;1(3):201–5.

[37] Tacon AGJ, Cowey CB. Protein and amino acid requirements. Fish energetics: new perspectives. Dordrecht: Springer Netherlands. 1985. p. 155–83.

[38] Wilson RP, Cowey CB. Amino acid composition of whole body tissue of rainbow trout and Atlantic salmon. Aquaculture. 1985;48(3–4):373–6.

[39] Hansen JM, Lazo JP, Kling LJ. A method to determine protein digestibility of microdiets for larval and early juvenile fish. Aquac Nutr. 2009;15(6).

[40] Tibbetts SM, Verreth JA, Lall SP. In vitro pH-Stat protein hydrolysis of feed ingredients for Atlantic cod, Gadus morhua. 2. In vitro protein digestibility of common and alternative feed ingredients. Aquaculture. 2011;319(3–4):407–16.

[41] Kwasek K, Gonzalez C, Wick M, Molinari GS, Wojno M. Fish muscle hydrolysate obtained using largemouth bass Micropterus salmoides digestive enzymes improves largemouth bass performance in its larval stages. PLoS One. 2021;16(12):e0261847. doi: 10.1371/journal.pone.0261847 34962940 PMC8714084

[42] Molinari GS, Wojno M, Terova G, Wick M, Riley H, Caminiti JT, et al. A Novel Approach in the Development of Larval Largemouth Bass Micropterus salmoides Diets Using Largemouth Bass Muscle Hydrolysates as the Protein Source. Animals (Basel). 2023;13(3):373. doi: 10.3390/ani13030373 36766261 PMC9913688

[43] Molinari GS, Wojno M, Terova G, Wick M, Riley H, Caminiti JT, et al. The Effect of the Species Source of Muscle and/or Digestive Enzymes on the Utilization of Fish Protein Hydrolysates as a Dietary Protein Source in First Feed for Larval Walleye (Sander vitreus). Animals (Basel). 2024;14(17):2493. doi: 10.3390/ani14172493 39272278 PMC11394479

[44] Ulloa PE, Medrano JF, Feijoo CG. Zebrafish as animal model for aquaculture nutrition research. Front Genet. 2014;5:313. doi: 10.3389/fgene.2014.00313 25309575 PMC4160086

[45] Dabrowski K, Miller M. Contested Paradigm in Raising Zebrafish (Danio rerio). Zebrafish. 2018;15(3):295–309. doi: 10.1089/zeb.2017.1515 29485943 PMC6037192

[46] Ramluckan K, Moodley KG, Bux F. An evaluation of the efficacy of using selected solvents for the extraction of lipids from algal biomass by the soxhlet extraction method. Fuel. 2014;116:103–8.

[47] Molinari GS, Wojno M, McCracken VJ, Kwasek K. The use of dipeptide supplementation as a means of mitigating the negative effects of dietary soybean meal on Zebrafish Danio rerio. Comp Biochem Physiol A Mol Integr Physiol. 2021;257:110958. doi: 10.1016/j.cbpa.2021.110958 33865992

[48] Perera E, Yúfera M. Soybean meal and soy protein concentrate in early diet elicit different nutritional programming effects on juvenile zebrafish. Zebrafish. 2016;13(1):61–9. doi: 10.1089/zeb.2015.1131 26716770

[49] Yossa R, Sarker PK, Karanth S, Ekker M, Vandenberg GW. Effects of dietary biotin and avidin on growth, survival, feed conversion, biotin status and gene expression of zebrafish Danio rerio. Comp Biochem Physiol B Biochem Mol Biol. 2011;160(4):150–8. doi: 10.1016/j.cbpb.2011.07.005 21839851

[50] Updike MS, Zerby H, Utrata KL, Lilburn M, Kaletunc G, Wick M. Proteins associated with thermally induced gelation of turkey breast meat. J Food Sci. 2006;71(9):E398-402.

[51] R Core Team. R: A language and environment for statistical computing. Vienna, Austria: R Foundation for Statistical Computing. 2022.

[52] Meyer G, Fracalossi DM. Estimation of jundiá (Rhamdia quelen) dietary amino acid requirements based on muscle amino acid composition. Scientia Agricola. 2005;62:401–5.

[53] Baras E, Jobling M. Dynamics of intracohort cannibalism in cultured fish. Aquac Res. 2002;33(7):461–79.

[54] Klemetsen A, Amundsen PA, Dempson JB, Jonsson B, Jonsson N, O’connell MF, et al. Atlantic salmon Salmo salar L., brown trout Salmo trutta L. and Arctic charr Salvelinus alpinus (L.): a review of aspects of their life histories. Ecol Freshw Fish. 2003;12(1):1–59.

[55] Nygaard H. Standard Norwegian fishmeal-and fishoil process. Heat treatment requirements. Nofima rapportserie. 2010.

[56] Hall GM. Fishmeal production and sustainability. Fish Processing: Sustainability and New Opportunities. 2010. p. 207–35.

[57] Liaset B, Lied E, Espe M. Enzymatic hydrolysis of by‐products from the fish‐filleting industry; chemical characterisation and nutritional evaluation. J Sci Food Agric. 2000;80(5):581–9.

[58] Vidotti RM, Viegas EM, Carneiro DJ. Amino acid composition of processed fish silage using different raw materials. Anim Feed Sci Technol. 2003;105(1–4):199–204.

[59] Hardy RW. Alternative marine sources of fish feed and farmed fish quality. Improving Farmed Fish Quality and Safety. Elsevier. 2008. p. 328–42. doi: 10.1533/9781845694920.2.328

[60] Olsen RL, Toppe J. Fish silage hydrolysates: Not only a feed nutrient, but also a useful feed additive. Trends Food Sci Technol. 2017;66.

[61] Kristinsson HG, Rasco BA. Fish protein hydrolysates: production, biochemical, and functional properties. Crit Rev Food Sci Nutr. 2000;40(1):43–81. doi: 10.1080/10408690091189266 10674201

[62] Espe M, Lied E. Fish silage prepared from different cooked and uncooked raw materials: chemical changes during storage at different temperatures. J Sci Food Agric. 1999;79(2):327–32.

[63] Siddik MA, Howieson J, Fotedar R, Partridge GJ. Enzymatic fish protein hydrolysates in finfish aquaculture: a review. Rev Aquac. 2021;13(1):406–30.

[64] Ryu B, Shin K-H, Kim S-K. Muscle protein hydrolysates and amino acid composition in fish. Mar Drugs. 2021;19(7):377. doi: 10.3390/md19070377 34210079 PMC8304736

[65] Wisuthiphaet N, Kongruang S, Chamcheun C. Production of fish protein hydrolysates by acid and enzymatic hydrolysis. J Med and Bioeng. 2015;4.

[66] Ghaly AE, Dave D, Budge S, Brooks MS. Fish spoilage mechanisms and preservation techniques. Am J Appl Sci. 2010;7(7):859.

[67] Silva JFX, Ribeiro K, Silva JF, Cahu TB, Bezerra RS. Utilization of tilapia processing waste for the production of fish protein hydrolysate. Anim Feed Sci Technol. 2014;196:96–106.

[68] Moyano FJ, Saénz de Rodrigáñez MA, Díaz M, Tacon AGJ. Application of in vitro digestibility methods in aquaculture: constraints and perspectives. Reviews in Aquaculture. 2014;7(4):223–42. doi: 10.1111/raq.12065

[69] Akiyama T, Oohara I, Yamamoto T. Comparison of essential amino acid requirements with A/E ratio among fish species. Fish Sci. 1997;63(6):963–70.

[70] Rustad T, Storrø I, Slizyte R. Possibilities for the utilisation of marine by‐products. Int J of Food Sci Tech. 2011;46(10):2001–14. doi: 10.1111/j.1365-2621.2011.02736.x

[71] Heffernan S, Giblin L, O’Brien N. Assessment of the biological activity of fish muscle protein hydrolysates using in vitro model systems. Food Chem. 2021;359:129852. doi: 10.1016/j.foodchem.2021.129852 33940471

[72] Ghaly AE, Ramakrishnan VV, Brooks MS, Budge SM, Dave D. Fish processing wastes as a potential source of proteins. Amino acids and oils: A critical review. J Microb Biochem Technol. 2013;5(4):107–29.

[73] Jobling M. Nutrient partitioning and the influence of feed composition on body composition. Food Intake in Fish. 2001;25(4):354–75.

[74] Jayathilakan K, Sultana K, Radhakrishna K, Bawa AS. Utilization of byproducts and waste materials from meat, poultry and fish processing industries: a review. J Food Sci Technol. 2012;49(3):278–93. doi: 10.1007/s13197-011-0290-7 23729848 PMC3614052

[75] Petricorena ZC. Chemical composition of fish and fishery products. Handbook of Food Chemistry. 2015. p. 28.

[76] Adatto I, Lawrence C, Krug L, Zon LI. The effects of intensive feeding on reproductive performance in laboratory zebrafish (Danio rerio). PLoS One. 2022;17(11):e0278302. doi: 10.1371/journal.pone.0278302 36445925 PMC9707780

[77] Kvåle A, Mangor-Jensen A, Moren M, Espe M, Hamre K. Development and characterisation of some intestinal enzymes in Atlantic cod (Gadus morhua L.) and Atlantic halibut (Hippoglossus hippoglossus L.) larvae. Aquaculture. 2007;264(1-4):457–68.

[78] Verri T, Kottra G, Romano A, Tiso N, Peric M, Maffia M, et al. Molecular and functional characterisation of the zebrafish (Danio rerio) PEPT1-type peptide transporter. FEBS Lett. 2003;549(1–3):115–22. doi: 10.1016/s0014-5793(03)00759-2 12914936

[79] Terova G, Robaina L, Izquierdo M, Cattaneo A, Molinari S, Bernardini G, et al. PepT1 mRNA expression levels in sea bream (Sparus aurata) fed different plant protein sources. Springerplus. 2013;2(1):17. doi: 10.1186/2193-1801-2-17 23449729 PMC3579422

[80] Ostaszewska T, Dabrowski K, Kamaszewski M, Grochowski P, Verri T, Rzepkowska M, et al. The effect of plant protein-based diet supplemented with dipeptide or free amino acids on digestive tract morphology and PepT1 and PepT2 expressions in common carp (Cyprinus carpio L.). Comp Biochem Physiol A Mol Integr Physiol. 2010;157(2):158–69. doi: 10.1016/j.cbpa.2010.06.162 20542130

[81] Kwasek K, Terova G, Wojno M, Dabrowski K, Wick M. The effect of dietary dipeptide lysine–glycine on growth, muscle proteins, and intestine PepT1 gene expression in juvenile yellow perch. Rev Fish Biol Fish. 2012;22:797–812.

[82] Sheng Z, Xu J, Zhang Y, Wang Z, Chen N, Li S. Dietary protein hydrolysate effects on growth, digestive enzymes activity, and expression of genes related to amino acid transport and metabolism of larval snakehead (Channa argus). Aquaculture. 2023;563:738896.

[83] Holt GJ. Larval fish nutrition. Hoboken, NJ: John Wiley & Sons. 2011.

[84] Borey M, Panserat S, Surget A, Cluzeaud M, Plagnes-Juan E, Herman A, et al. Postprandial kinetics of gene expression of proteins involved in the digestive process in rainbow trout (O. mykiss) and impact of diet composition. Fish Physiol Biochem. 2016;42(4):1187–202. doi: 10.1007/s10695-016-0208-4 26920536

[85] Cai Z, Li W, Mai K, Xu W, Zhang Y, Ai Q. Effects of dietary size-fractionated fish hydrolysates on growth, activities of digestive enzymes and aminotransferases and expression of some protein metabolism related genes in large yellow croaker (Larimichthys crocea) larvae. Aquaculture. 2015;440:40–7.

[86] Wu D, Zhou L, Gao M, Wang M, Wang B, He J. Effects of stickwater hydrolysates on growth performance for yellow catfish (Pelteobagrus fulvidraco). Aquaculture. 2018;488:161–73.

[87] Chen X, Wang J, Qian L, Gaughan S, Xiang W, Ai T, et al. Domestication drive the changes of immune and digestive system of Eurasian perch (Perca fluviatilis). PLoS One. 2017;12(3):e0172903. doi: 10.1371/journal.pone.0172903 28257494 PMC5336236

[88] Palińska-Żarska K, Woźny M, Kamaszewski M, Szudrowicz H, Brzuzan P, Żarski D. Domestication process modifies digestion ability in larvae of Eurasian perch (Perca fluviatilis), a freshwater Teleostei. Sci Rep. 2020;10(1):2211. doi: 10.1038/s41598-020-59145-6 32042003 PMC7010758

[89] Montero D, Moyano FJ, Carvalho M, Sarih S, Fontanillas R, Zamorano MJ, et al. Nutritional innovations in superior gilthead seabream (Sparus aurata) genotypes: implications in the utilization of emerging new ingredients through the study of the patterns of secretion of digestive enzymes. Aquaculture. 2023;577:739958.

[90] Kaushik S, Georga I, Koumoundouros G. Growth and body composition of zebrafish (Danio rerio) larvae fed a compound feed from first feeding onward: toward implications on nutrient requirements. Zebrafish. 2011;8(2):87–95. doi: 10.1089/zeb.2011.0696 21663450

[91] Farias M, Certal AC. Different feeds and feeding regimens have an impact on zebrafish larval rearing and breeding performance. Int J Marine Biol Res. 2016;1(1):1–8.

[92] Printzi A, Koumoundouros G, Fournier V, Madec L, Zambonino-Infante J-L, Mazurais D. Effect of Early Peptide Diets on Zebrafish Skeletal Development. Biomolecules. 2023;13(4):659. doi: 10.3390/biom13040659 37189406 PMC10135642

[93] Curnow J, King J, Bosmans J, Kolkovski S. The effect of reduced artemia and rotifer use facilitated by a new microdiet in the rearing of barramundi Lates calcarifer (Bloch) larvae. Aquaculture. 2006;257(1–4):204–13.

[94] Canavate JP, Fernández-Dıaz C. Influence of co-feeding larvae with live and inert diets on weaning the sole Solea senegalensis onto commercial dry feeds. Aquaculture. 1999;174(3–4):255–63.

[95] Robison BD, Rowland W. A potential model system for studying the genetics of domestication: behavioral variation among wild and domesticated strains of zebra danio (Danio rerio). Can J Fish Aquat Sci. 2005;62(9):2046–54.

[96] Nen PH, Chheng P, So N, Hien TT, Tam BM, Egna HI. Performance of domesticated (Vietnamese) versus non-domesticated (Cambodian) snakehead, Channa striata (Bloch 1793) with regard to weaning onto pellet feed. Asian Fish Sci. 2018;31(3):209–17.

[97] Milla S, Pasquet A, El Mohajer L, Fontaine P. How domestication alters fish phenotypes. Rev Aquac. 2021;13(1):388–405.

[98] Balon EK. About the oldest domesticates among fishes. J Fish Biol. 2004;65:1–27.

[99] Teletchea F. Domestication level of the most popular aquarium fish species: is the aquarium trade dependent on wild populations. Cybium. 2016;40(1):21–9.

[100] Carvalho AP, Escaffre AM, Oliva Teles A, Bergot P. First feeding of common carp larvae on diets with high levels of protein hydrolysates. Aquac Int. 1997;5:361–7.

[101] Teletchea F. Fish domestication in aquaculture: 10 unanswered questions. Anim Front. 2021;11(3):87–91. doi: 10.1093/af/vfab012 34158993 PMC8214440

[102] Lawrence C, Adatto I, Best J, James A, Maloney K. Generation time of zebrafish (Danio rerio) and medakas (Oryzias latipes) housed in the same aquaculture facility. Lab Anim (NY). 2012;41(6):158–65. doi: 10.1038/laban0612-158 22614091

[103] Suurväli J, Whiteley AR, Zheng Y, Gharbi K, Leptin M, Wiehe T. The Laboratory Domestication of Zebrafish: From Diverse Populations to Inbred Substrains. Mol Biol Evol. 2020;37(4):1056–69. doi: 10.1093/molbev/msz289 31808937 PMC7086173

[104] Tamotsu S, Fujita S, Ogata Y, Kimura I. Postmortem autolysis of juvenile sardines during cold storage. Nippon Suisan Gakkaishi. 2018;84(1):103–10.

[105] Nankervis L, Southgate PC. Enzyme and acid treatment of fish meal for incorporation into formulated microbound diets for barramundi (Lates calcarifer) larvae. Aquaculture Nutr. 2009;15(2):135–43.

[106] Cahu C, Rønnestad I, Grangier V, Infante JZ. Expression and activities of pancreatic enzymes in developing sea bass larvae (Dicentrarchus labrax) in relation to intact and hydrolyzed dietary protein; involvement of cholecystokinin. Aquaculture. 2004;238(1–4):295–308.

[107] Liu F, Mai KS, Ai QH, Duan QY, Xu W, Tan BP. Effects of dietary fish protein hydrolysate levels on growth, survival and body composition of larvae in Pseudosciaena crocea. J Fish China. 2006;30(4):502–8.

