# Supplementary figures and images for: New approach to the development of tailor-made feed for fish larvae using Zebrafish Danio rerio as a model
Source: PLoS One. 2025 Jun 24;20(6):e0326665. doi: 10.1371/journal.pone.0326665 (PMC12186918; doi:10.1371/journal.pone.0326665)

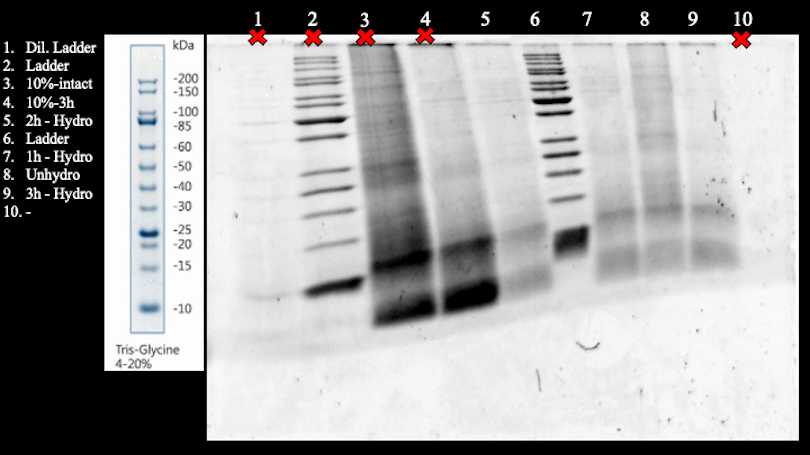

Supplement: S2 File — (TIF) [file pone.0326665.s002.tif]
